# Physiological and Phylogenetic Characterization of *Rhodotorula diobovata* DSBCA06, a Nitrophilous Yeast

**DOI:** 10.3390/biology7030039

**Published:** 2018-06-30

**Authors:** Enrico Civiero, Manuela Pintus, Claudio Ruggeri, Elena Tamburini, Francesca Sollai, Enrico Sanjust, Paolo Zucca

**Affiliations:** Dipartimento di Scienze Biomediche, Università degli Studi di Cagliari, SP 1 Km 0,700, 09042 Monserrato (CA), Italy; civieroenrico.81@gmail.com (E.C.); manuela.pintus@gmail.com (M.P.); rugg.claudio@gmail.com (C.R.); etamburini@unica.it (E.T.); sollai@unica.it (F.S.); pzucca@unica.it (P.Z.)

**Keywords:** nitrogen, nitrite, bioremediation, wastewater, eutrophication

## Abstract

Agriculture and intensive farming methods are the greatest cause of nitrogen pollution. In particular, nitrification (the conversion of ammonia to nitrate) plays a role in global climate changes, affecting the bio-availability of nitrogen in soil and contributing to eutrophication. In this paper, the *Rhodotorula diobovata* DSBCA06 was investigated for growth kinetics on nitrite, nitrate, or ammonia as the sole nitrogen sources (10 mM). Complete nitrite removal was observed in 48 h up to 10 mM initial nitrite. Nitrogen was almost completely assimilated as organic matter (up to 90% using higher nitrite concentrations). The strain tolerates and efficiently assimilates nitrite at concentrations (up to 20 mM) higher than those previously reported in literature for other yeasts. The best growth conditions (50 mM buffer potassium phosphate pH 7, 20 g/L glucose as the sole carbon source, and 10 mM nitrite) were determined. In the perspective of applications in inorganic nitrogen removal, other metabolic features relevant for process optimization were also evaluated, including renewable sources and heavy metal tolerance. Molasses, corn, and soybean oils were good substrates, and cadmium and lead were well tolerated. Scale-up tests also revealed promising features for large-scale applications. Overall, presented results suggest applicability of nitrogen assimilation by *Rhodotorula diobovata* DSBCA06 as an innovative tool for bioremediation and treatment of wastewater effluents.

## 1. Introduction

In the last decades, agriculture and intensive farming increased the use of inorganic fertilizers leading to huge health and economic benefits but also to severe input of polluting nitrogen compounds into the environment.

Ammonium is the most easily assimilated inorganic nitrogen form. However, nitrification causes the loss of enormous amounts of nitrogen in the most oxidized forms (nitrite and nitrate), affecting global climate changes and increasing environmental pollution [1]. Enrichment of a water body with an excess amount of nutrients causes, for instance, eutrophication [2]. Nitrite is also toxic to humans, since it oxidizes the ferroheme in red blood cells and as a consequence prevents the oxygen carrying by hemoglobin [3]. Furthermore, nitrite can be transformed to genotoxic nitrosamines by some bacteria [4].

Industrialized countries counteracted the problem with laws restricting the use of simple ionic nitrogen compounds [5]. For instance, the European Commission issued the *Nitrates Directive* (91/676/EEC) and the *Water Framework Directive* (2000/60/EC) to protect ground and surface waters against polluting nitrogen compounds from agricultural sources. Conventional technologies for nitrogen removal from wastewater effluents and for bioremediation of surface and ground waters are based on phytoremediation treatments as well as microbial denitrification, finally leading to molecular nitrogen [6,7]. An innovative strategy to limit nitrogen pollution refers to the study of physiological and molecular mechanisms involved in nitrate and nitrite assimilation by microorganisms, converting them back into ammonia form [8,9].

Many prokaryotic and eukaryotic microorganisms are capable of utilizing nitrate as the sole nitrogen source [6], reducing it back to ammonium by the consecutive catalysis of nitrate and nitrite reductases [8]. In yeasts, the use of nitrate and nitrite is restricted to relatively few species of different genera [8]. The physiology and enzymology of nitrate assimilation have been extensively studied in nitrophilous yeasts, such as *Candida*, and *Rhodotorula* [10], as well as *Wickerhamomyces* and *Ogataea* (formerly *Hansenula*) [11]*.* Nitrophilous yeasts exhibit rapid growth at concentrations higher than 10 mM nitrate-nitrogen, while similar nitrate concentrations are usually toxic, inhibiting yeast growth [12]. On the contrary, tolerance to high nitrite concentrations has been rarely documented. Nitrite assimilation has been investigated in *Candida nitratophila*, which uptakes nitrite at 1 mM [12]. More recently, Vigliotta et al. [13] have demonstrated the ability of a yeast strain of *Debaryomyces hansenii* to tolerate nitrite up to about 20 mM and to utilize nitrite, but not nitrate, as a sole nitrogen source, a property that was enhanced in microaerophilic environment.

In this work, we performed the isolation and characterization of a novel nitrophilous *Rhodotorula* strain. The ability to assimilate nitrogen compounds and to tolerate high nitrite concentrations was investigated. Moreover, other metabolic features relevant for process optimization were evaluated, including carbon sources, pH optimum, and heavy metal tolerance. In the last decades, *Rhodotorula* species have found increasing applications in the production of biotechnologically interesting products, such as carotenoids, for pharmaceutical, chemical, food, and feed industries [14,15] as well as in bioremediation of oil-polluted environments and heavy metal biosorption [14,16,17,18]. Nevertheless, to our knowledge, this is the first report on the ability of a *Rhodotorula* yeast to efficiently assimilate nitrite and well tolerate concentrations up to 20 mM nitrite. Presented results suggests its applicability as a tool for inorganic nitrogen removal in bioremediation or wastewater treatments.

## 2. Materials and Methods

### 2.1. Chemicals

All the reagents used were of the best grade available and were used as purchased without further purification.

Spectrophotometric analyses were carried out with UltroSpec 2100pro (Amersham Bioscience, Milan, Italy). Concentrations of substrates and composition of industrial carbon sources were checked by UV-HPLC and GC-MS, using previously described methods [19,20].

### 2.2. Strain Isolation

The basal composition of the Yeast Nitrogen Base (YNB) agar medium was as follows (per liter): 50 mL of buffer potassium phosphate (1 M, pH 7), 10 mL of vitamins and oligoelements solution (0.2 mg/L, folic acid 0.2 mg/L, copper sulfate 4 mg/L, potassium iodide 10 mg/L, *p*-amino benzoic acid 20 mg/L, calcium pantothenate 40 mg/L, inositol 200 mg/L, niacin 40 mg/L, pyridoxine 40 mg/L, riboflavin 20 mg/L, thiamine hydrochloride 40 mg/L, boric acid 50 mg/L, ferric chloride 20 mg/L, manganese sulfate 40 mg/L, sodium molybdate 20 mg/L, zinc sulfate 40 mg/L), potassium dihydrogen phosphate 0.2 g, sodium chloride 0.02 g, magnesium sulfate 0.1 g, calcium chloride 0.02 g, agarose 15 g [21].

For the selection of nitrate tolerant yeasts, the basal YNB medium was supplemented with glucose 20.0 g/L, as the sole carbon source, and 20 mM NaNO_3_, as a sole nitrogen source. The Petri dishes were exposed overnight to open air in the countryside of the campus area in Monserrato (Cagliari, Italy), and incubated at 25 °C for 72 h. Among the most rapidly growing colonies, the pink ones were obtained in pure culture by repeated streaks. The isolate was named DSBCA06 and included in our Departmental collection. The strain was maintained at 25 °C on Glucose Yeast Peptone (GYP) agar medium, containing 20 g/L glucose, 10 g/L peptone from casein, 10 g/L yeast extract, 50 mM potassium phosphate buffer, pH 6 (see Supplementary Material). The strain was stored at −20 °C in a solution made from 60% GYP medium and 40% glycerol. When required, the strain was isolated on GYP agar and isolated colonies used for inoculating liquid cultures in GYP medium.

### 2.3. Molecular Characterization

The Wizard genomic DNA purification kit (Promega, Madison, WI, USA) was used for genomic DNA extraction, according to the manufacturer’s instructions. Amplification and sequencing of the internal transcribed spacers (ITS1-5.8S-ITS2) as well as the D1/D2 variable regions at the 5′ end of the large subunit (LSU) rRNA gene were performed by an external service (BMR Genomics, Padua, Italy) [22,23]. The obtained sequence was deposited in Genbank under the accession number MG777534. The sequence was analyzed with the basic local alignment search tool (BLAST). For taxonomic assignment, the sequences of the closely related validly published type strains were retrieved from Genbank. The software Clustal W was used for sequence alignment and a phylogenetic tree was contracted using MEGA 7 [24] by applying the neighbor-joining method [25].

### 2.4. Characterization and Optimization of Catabolic Features

Liquid cultures were prepared in GYP medium and incubated 18 h at 25 °C in a rotary shaker at 150 rpm. The cells were removed by centrifugation, washed twice in sodium chloride 0.9% *w*/*v*, and suspended in YNB minimal medium. The cultures were prepared in 100 mL flasks containing 25 mL of YNB minimal medium and inoculated to an initial OD_600_ of 0.050. Unless otherwise stated, 20 g/L glucose was the sole carbon source. Flasks were closed with cellulose caps to allow proper aeration. Cultures were incubated at 25 °C in a rotary shaker at 150 rpm. Three different nitrogen sources were singularly evaluated: 10 mM NaNO_2_, 10 mM NaNO_3_, and 5 mM (NH_4_)_2_SO_4_. All the samples were assayed in biological double, and all data were reported as the mean of at least three independent measurements.

Different carbon and energy sources were included in the study: glucose, sucrose, fructose, maltose, mannose, mannitol, lactose, galactose, acetate, glycerol, gluconate, ethanol, and citrate. Each selected carbon source was sterilized by filtration and supplied at an initial concentration of 2% *w*/*v*. Several renewable carbon sources were also evaluated: beet molasses 2% *w*/*v*, olive mill wastewater (OMW, from local mills) 2% *w*/*v*, neutralized black liquor (BL), obtained from a paper factory 1% *v*/*v*, cork (*Quercus suber* bark) factory wastewater (CFW), collected in Giara di Tuili (Sardinia, Italy) 2% *w*/*v*, soybean oil 2% *v*/*v*, corn oil 2% *v*/*v*, paraffin oil 2% *v*/*v*.

The influence of pH was studied using 50 mM potassium phosphate buffer for pH 6 and pH 7, 50 mM sodium acetate buffer for pH 4 and 5, and 50 mM sodium pyrophosphate buffer for pH 8 and 9.

The limiting concentrations defined from Italian laws D.lgs. 152/2006 and 2000/60/EC soil values limits (Table 1) were used to prepare liquid cultures containing some heavy metals (Cd, Co, Hg, Ni, and Pb).

### 2.5. Nitrogen Assimilation

Dry weight measurements were carried out with cellulose nitrate filters (Sartorius Stedim, 0.45 μm, Göttingen, Germany). Filters were kept at 100 °C for one hour and weighed before culture filtration, using a Millipore system (All-Glass Filter Holder Assembly with funnel, fritted base, cap, clamp, 47 mm; Merck, Darmstadt, Germany). Then, filters were kept for 24 h at 100 °C and rapidly weighed. Sample weights were determined by difference.

An oxidant solution based on potassium peroxydisulfate was used to quantify nitrogen present in cells (1 g potassium peroxydisulfate, 0.225 g boric acid, and 0.105 g sodium hydroxide, in a final volume of 15 mL). Aliquots of 1.5 mg cells (dry weight) were treated in a glass tube with 0.7 mL of oxidant solution and glass balls (diameter 0.45–0.5 mm) and shaken to ease breaking of cell walls and membranes. Then, 2.5 mL water was added and samples were autoclaved at 120 °C for 30 min.

After this treatment all nitrogen present in the original sample was oxidized to nitrate and quantified using Griess method (*vide ultra*).

Nitrite, nitrate, and ammonium were also quantified in the cell-free filtrate and in the cell extract for determining initial, final, as well as organic nitrogen in culture supernatants and in microbial biomass.

### 2.6. Analytic Determinations

Nitrite and nitrate were determined using a previously described method [26], exploiting the reaction between nitrite, sulfanilamide (SULF), and *N*-(1-naphthyl)ethylenediamine dihydrochloride (NEDD) (also called Griess reagent) producing a pink-purple azo dye.

Nitrite assay was performed incubating 400 µL distilled H_2_O, 200 µL SULF (2% *w*/*v* in HCl 5%), 200 µL NEDD (0.1% *w*/*v* in H_2_O), 400 µL sample. After 45 min in the dark, absorbance was read at 540 nm.

Nitrate measurement requires an extra reaction: the previous reduction to nitrite using VCl_3_ under acidic conditions [27].

Assay preparation was the same as above, except for the substitution of H_2_O with 400 µL VCl_3_ solution (400 mg VCl_3_ in 50 mL of HCl 1 M, prepared and used within 72 h).

Ammonia was determined with the method of Emmet, using an oxidizing solution, prepared by adding 25 mL of 5% sodium hypochlorite (active chlorine) to 100 mL of a solution containing 20% *w*/*v* trisodium citrate dihydrate and 0.01 *w*/*v* sodium hydroxide.

The analysis required the incubation of 1 mL sample, 4 mL H_2_O, 0.2 mL phenol alcoholic solution (10% *v*/*v*), 0.2 mL sodium nitroprusside solution (5 g/L), and 0.5 mL oxidizing solution. Photometric measures were carried out after 1 h of incubation. The absorbance of the arising indophenol dye was read at 630 nm.

Glucose was quantified by the enzymatic kit, using horseradish peroxidase (HRP) and glucose oxidase (GOX). During the assay, samples were incubated in a 0.1 M potassium phosphate buffer pH 6, 1 M), 1 EU of HRP, 2 EU of GOX, 0.2 mM ABTS (2,2′-azino-*bis*(3-ethylbenzothiazoline-6-sulfonic acid)). Analysis was made measuring absorbance variation at 420 nm for 1 min. A standard curve was prepared using glucose solutions.

### 2.7. Process Scale-Up

An Applikon (Applikon, Delft, The Netherlands) bio-reactor (total volume 2.3 L) was used equipped with Bio controller ADI 1032 (Applikon, Delft, Netherlands) for temperature, pH, and oxygen control, and with Stirrer Controller P100 ADI 1032 (Applikon, Delft, Netherlands) for the control of the stirring engine. Working volume in the fermenter was 1 L.

## 3. Results and Discussion

### 3.1. Phylogenetic Characterization

**Figure 1:**
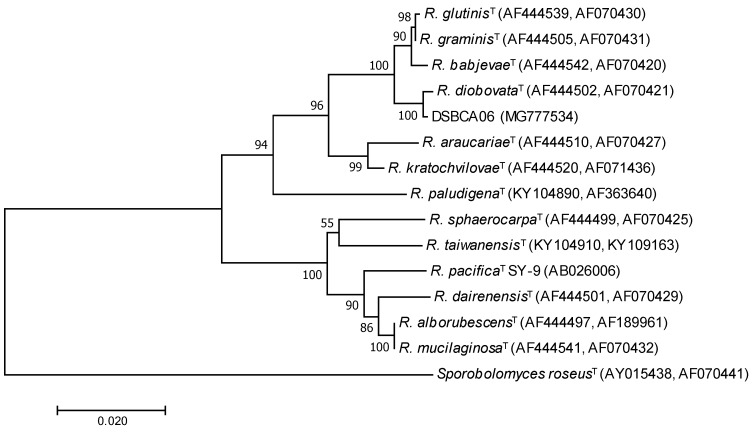
Unrooted phylogenetic tree based on the comparisons of the internal transcribed spacer (ITS) regions and D1/D2 variable domains of the large subunit (LSU) rRNA gene showing the position of the strain DSBCA06 (characterized in this work) and the type strains of selected related species. The tree was constructed with a total of 1146 positions, using a neighbor-joining distance matrix. The scale bar indicates substitutions per nucleotide position. The Bootstrap values are indicated at the nodes. The GenBank accession numbers are reported in brackets.

The air isolate DSBCA06 was selected for its resistance to nitrate at 20 mM. Analysis of the nucleotide sequence of the internal transcribed spacer (ITS) regions and D1/D2 variable domains of the LSU rRNA gene demonstrated the strain DSBCA06 exhibits the highest sequence similarity (99.7%) with *Rhodotorula diobovata* (formerly *Rhodosporidium diobovatum*). A phylogenetic tree was generated showing the strain DSBCA06 was phylogenetically closely related to the species *R. graminis*, *R. glutinis*, and *R*. *babjevae*, which form a distinct cluster with *R. diobovata* in the genus *Rhodotorula* (Figure 1). Members of these species have been studied for nitrate assimilation [28,29] and extensively investigated as oleaginous yeasts for the production of carotenoids [18,30] and fatty acids [31].

### 3.2. Comparison of Nitrogen Sources

**Figure 2:**
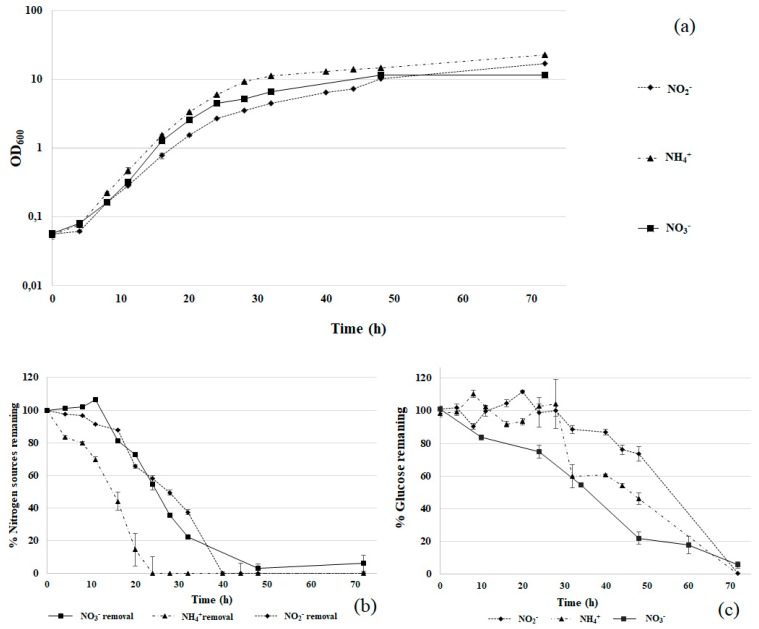
*R. diobovata* DSBCA06 growth in Yeast Nitrogen Base (YNB) + glucose 2% with different nitrogen sources: 10 mM NaNO_3_, 10 mM NaNO_2_, and 5 mM (NH_4_)_2_SO_4_ (panel (**a**)). Nitrogen and glucose removal are reported in panels (**b**,**c**). OD: optical density at 600 nm.

The ability of *R. diobovata* DSBCA06 to assimilate different nitrogen sources was investigated in liquid cultures. This metabolic property is crucial in the perspective of its application in wastewater treatments as well as in bioremediation of surface and ground waters. Different nitrogen salts were evaluated (10 mM NaNO_3_, 10 mM NaNO_2_, and 5 mM (NH_4_)_2_SO_4_) and the corresponding growth curves are reported in Figure 2.

Ammonium led to higher biomass production than the other tested nitrogen sources, but the specific growth rates (μ) were quite similar: 0.220 h^−1^ for ammonium, 0.224 h^−1^ for nitrate, and 0.200 h^−1^ for nitrite. This finding suggests that all the three nitrogen sources are well tolerated by the studied strain.

Nitrogen assimilation followed a similar pattern. Ammonium was the fastest removed nitrogen source (in 20 h no ammonium was detectable in the medium). This result is probably due to a simple assimilation of this nitrogen source, compared with nitrate and nitrite, requiring specific enzymatic activities for the reduction of oxidized nitrogen forms to ammonium nitrogen. However, complete removal of nitrate and nitrite was also achieved after 40–50 h (Figure 2b). After the complete removal from the medium, the growth did not stop, suggesting that *R. diobovata* DSBCA06 is able to store a certain extent of nitrogen within the cell, using it at a later time.

On the whole, all the three nitrogen sources were assimilated by *R. diobovata* DSBCA06, therefore making the studied strain a potential candidate in the development of wastewater treatment processes or novel bioremediation technology for nitrogen contaminated surface and ground waters. Currently, several technologies based on bacterial metabolism are reported for biological nitrogen removal [7], but only rarely with assimilation mechanism [32]. On the contrary, yeasts did not yet find any large-scale application, as well as such assimilation mechanism here reported.

At a laboratory scale, nitrate and ammonium assimilation have been largely studied in different yeast genera [8,9,33,34]. Even representing the more toxic nitrogen form, nitrite tolerance and assimilation is quite less investigated in yeast. Based on the ability of the investigated strains to efficiently grow and assimilate at high nitrite concentration (10 mM), the study was focused on nitrite tolerance and assimilation.

### 3.3. Physiological Characterization and Optimization of Growth Conditions

**Figure 3:**
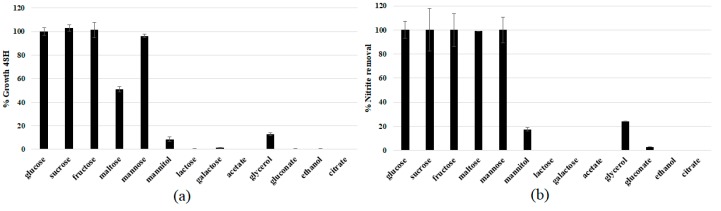
*R. diobovata* DSBCA06 relative 48 h growth in YNB + 10 mM NaNO_2_ (**a**); using different carbon sources at (2% *w*/*v*), and corresponding nitrite removed (**b**).

In order to identify the best carbon and energy sources sustaining the growth of the strain DSBCA06, culture broths were prepared with different substrates. Fructose, glucose, mannose, galactose (monosaccharides), sucrose, maltose, lactose (disaccharides), mannitol, glycerol (sugar derived polyols), but also organic acids (acetate, citrate, gluconate), and ethanol, were included in the screening, and the results reported in Figure 3. Lactose, galactose, acetate, citrate, and gluconate are not substrates at all, resulting in negligible growth after 48 h. On the contrary, the highest growth rate was observed using simple sugars such as glucose, fructose, sucrose, and mannose, showing an almost complete nitrite removal in 48 h. Glycerol allowed only about 15% growth (using glucose as the reference), although in the literature this substrate is often used as a carbon source for other strains belonging to the *Rhodotorula* genus [35,36,37,38]. The obtained results suggest the use of glucose as the carbon source for the studied strain, essentially for economic concerns (it is the most inexpensive substrate among the ones with the most promising results).

The effect of pH on microbial growth was also investigated, since pH directly influences metabolism and secondary metabolites production. Different buffers were used: sodium acetate buffer for pH 4 and 5, potassium phosphate buffer for pH 6 and 7, and sodium pyrophosphate buffer for pH 8 and 9. Negligible growth was observed outside pH 6–7, and nitrite removal was highest around neutrality, suggesting neutrality as the pH for further optimization studies (see Supplementary Material).

The use of raw materials, as low-cost growth substrates, is a key strategy to affect economic feasibility of microbial processes, accounting for about 10–30% of the total production costs in most biotechnological processes [39]. The ability of *R. diobovata* DSBCA06 to grow on a variety of cheap renewable substrates was then studied (see Supplementary Material), ranging from edible and non-edible vegetable and mineral oils, to industrial wastes (black liquor, molasses). The use of cheap raw materials and wastes could, in fact, reduce processing costs for possible large-scale industrial application of the strain. Moreover, preliminary data on the anabolic features of *R. diobovata* DSBCA06 (data not shown) suggest that inorganic-nitrogen-contaminated wastewater treatments or ex situ bioremediation could be coupled with the production of high value-added metabolites, such as lipids for biodiesel production, and carotenoids.

*R. diobovata* DSBCA06 was able to grow efficiently on molasses, corn, and soybean oils (growth 60–70% of control in 72 h), but also on olive mill wastewater (about 40% control). However, no significant biomass increase after 3 days was observed using black liquor, paraffin, or on cork factory wastewaters.

### 3.4. Nitrite Tolerance

**Figure 4:**
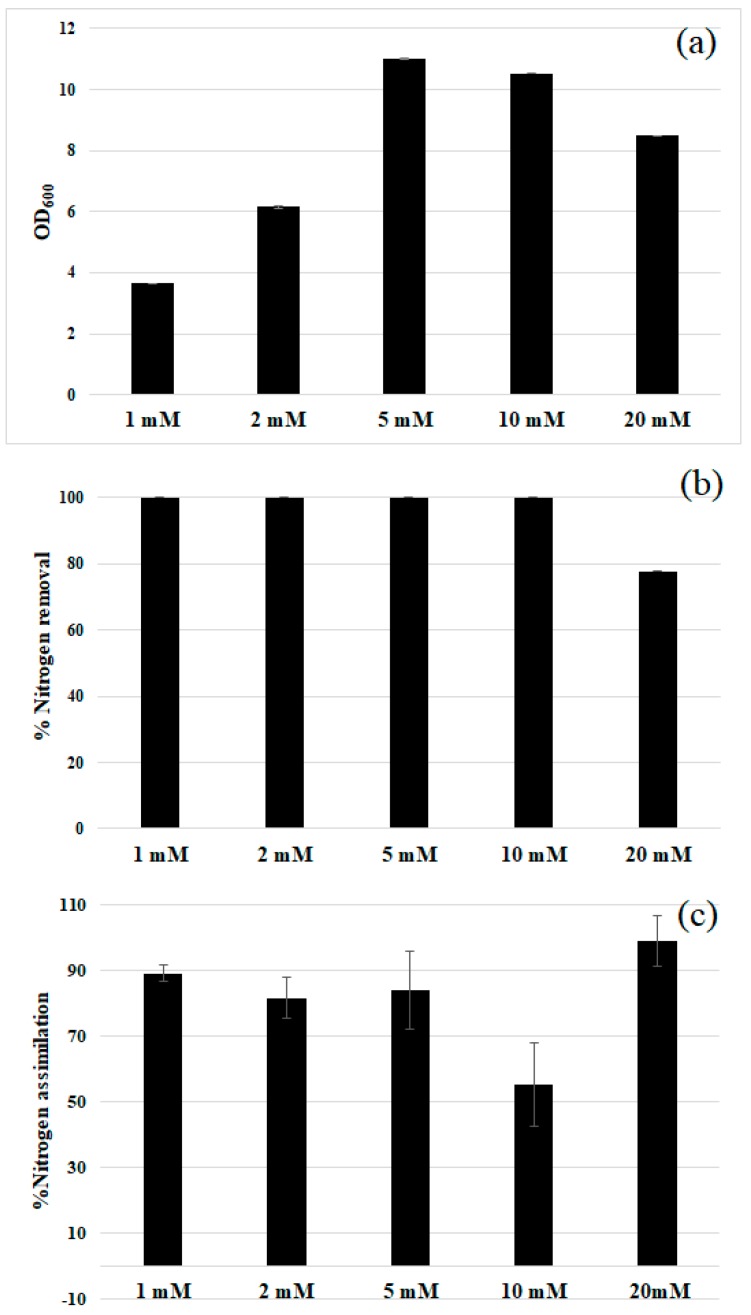
*R. diobovata* DSBCA06 tolerates up to 20 mM nitrite, with small difference in 48 h growth (**a**); Nitrite removal for each of the tested concentrations in 48 h was almost complete (**b**); showing that it was almost completely assimilated in organic compounds (**c**).

The ability of the studied strain to tolerate increasing nitrite concentrations was also evaluated (Figure 4). The strain showed an ideal growth in the range 5–10 mM, but up to 20 mM nitrite was well tolerated. Higher concentrations led to a complete growth inhibition. Almost complete nitrite removal was observed in 48 h up to 10 mM initial nitrite (panel (b), Figure 4). Whereas, in the presence of 20 mM nitrite 80% of removal was obtained. The removed nitrite was almost completely assimilated as organic nitrogen (panel (c), Figure 4), ruling out any denitrifying activity. According to these results, 10 mM was considered as the optimal initial nitrite concentration for the rest of the study.

These data show that *R. diobovata* DSBCA06 tolerates nitrite concentrations higher than the usual level present in typical polluted environments (the limit level in water is about 0.06 mM [40]). This toxic nitrogen form is then converted into non-toxic organic compounds, that in turn could have potential secondary applications (i.e., fertilizers or animal feed). The strain tolerance to nitrite is higher if compared to what is previously reported for some bacteria [41] and other yeasts [12]. Its efficiency is quite similar to that previously found for *Aspergillus carneus* [42]. Recently, Vigliotta et al. showed a similar nitrite tolerance for *Debaryomyces hansenii* TOB-Y7; however, in this case almost complete removal was obtained in about 12 days [13]. Accordingly, our results are promising in the perspective of application of the strain DSBCA06 as an innovative tool for the nitrogen removal in wastewater treatments and bioremediation.

### 3.5. Heavy Metal Tolerance

One important feature for the exploitation of the investigated strain as a tool for nitrogen removal in wastewater treatment and bioremediation can be the tolerance to heavy metals, since contaminated environmental matrices and industrial effluents are frequently co-contaminated by inorganic nitrogen compounds and metals [43]. Heavy metals have toxic effects on cells but several bacteria and yeasts can tolerate elevated heavy metal concentrations [44,45].

**Figure 5:**
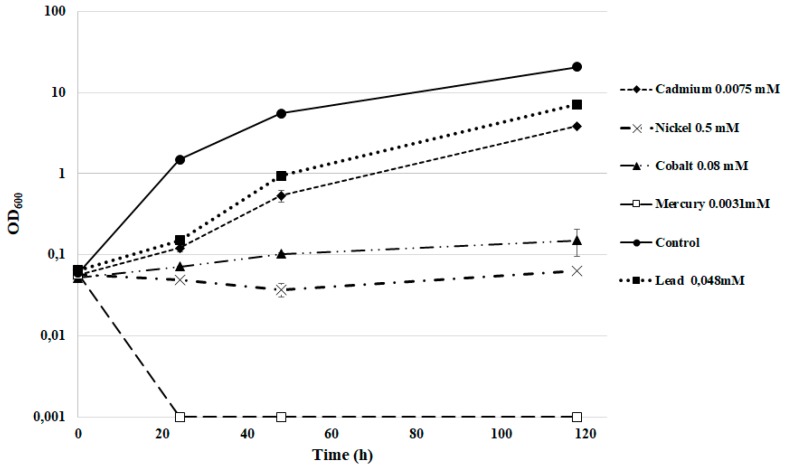
*R. diobovata* DSBCA06 growth in YNB in the presence of polluting heavy metals.

Many yeasts present both high resistance to heavy metals and also the ability of bioaccumulation in vacuoles. This ability shows a high variability depending on several environmental and biological factors [46]. In this perspective, we tested the strain DSBCA06 for its ability to grow in the presence of several heavy metals, using the concentrations used as limits by the current Italian law. Only mercury completely inhibits the yeast growth, whereas nickel and cobalt led to a very slow growth. In YNB medium with elevated concentrations of nitrite (10 mM), *R. diobovata* DSBCA06 was able to grow in the presence of cadmium or lead (Figure 5), showing only a minimal decrease in the rate of growth. A similar tolerance pattern has been well described for other *Sporobolomyces* and *Rhodotorula* spp. [47,48,49,50]. With an eye to the development of large-scale bioremediation and wastewater treatment processes, these reported data are very promising.

### 3.6. Nitrite Removal at Bioreactor Scale

**Figure 6:**
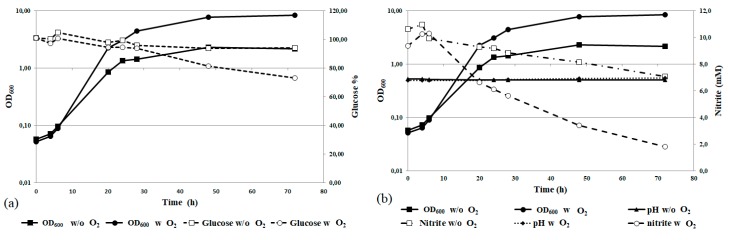
*R. diobovata* DSBCA06 growth in YNB using a bioreactor, with and without aeration. Glucose removal is reported in panel (**a**); whereas nitrite removal and pH measurements in (**b**).

Preliminary experiments of scaling-up in 3 L batch bioreactors were performed to evaluate the feasibility of large-scale nitrogen removal processes. The optimized growth conditions were used, and forced aeration was tested as well. The results are reported in Figure 6.

Scaling-up was performed without significant modification of the strain growth pattern, as demonstrated by similar pattern of the growth curves. However, forced aeration seems to be a key requirement, in accordance with the fact that *Rhodotorula* spp. are aerobic microorganisms. In absence of forced aeration, in fact, significant limitation of biomass production, decrease in the rate of nitrite removal, and glucose consumption were observed (Figure 6b).

## 4. Conclusions

The nitrophile yeast *Rhodotorula diobovata* DSBCA06 has been identified and studied to define its nitrogen-based metabolism, optimize the assimilation of nitrogen compounds, and evaluate the potential of the strain for inorganic nitrogen removal in wastewater treatment and bioremediation. The described process could be applied to reduce oxidized nitrogen polluting compounds converting them into biomass, less environmentally impacting and with potential application as fertilizers or as animal feed.

Overall, *R. diobovata* DSBCA06 shows promising features for the future development of economically efficient industrial-scale biotechnological processes being supported by low cost renewable substrates.

## Figures and Tables

**Table 1:** Limits for heavy metal concentrations in green areas (Italian law D.lgs. 152/06).

Metal	mg/kg	mM
Cadmium acetate	2	0.0075
Cobalt acetate	20	0.08
Mercury acetate	1	0.0031
Nickel chloride	120	0.5
Lead acetate	100	0.264

## Supplementary Materials

The following are available online at http://www.mdpi.com/2079-7737/7/3/39/s1, Figure S1: Colonies of *R. diobovata* DSBCA06 in YNB and GYP media; the same yeast cells observed at optic microscope, Figure S2: Influence of pH on *R. diobovata* DSBCA06 growth. In panel (a) initial and final pH values tested. Panels (b) and (c) report the corresponding growth and nitrite removal 48 h, Figure S3: *R. diobovata* DSBCA06 growth in YNB using different renewable wastes as carbon sources. (OMW: olive mill wastewater; CFW: cork factory wastewater; BL: neutralized black liquor).
