# Supplementary material for: Physiological and Phylogenetic Characterization of Rhodotorula diobovata DSBCA06, a Nitrophilous Yeast
Source: Biology (Basel). 2018 Jun 30;7(3):39. doi: 10.3390/biology7030039 (PMC6163231; doi:10.3390/biology7030039)
Supplement: Supplementary file 1 [file biology-07-00039-s001.pdf]

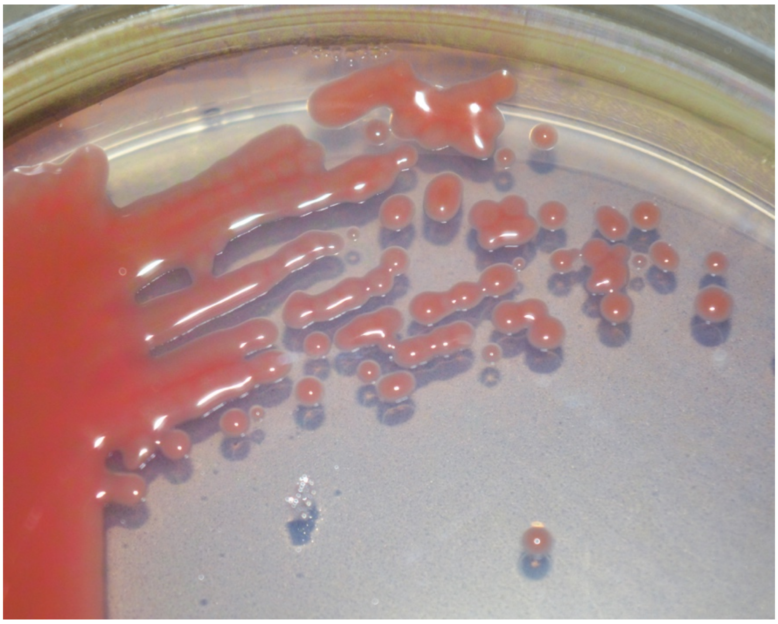

(a)

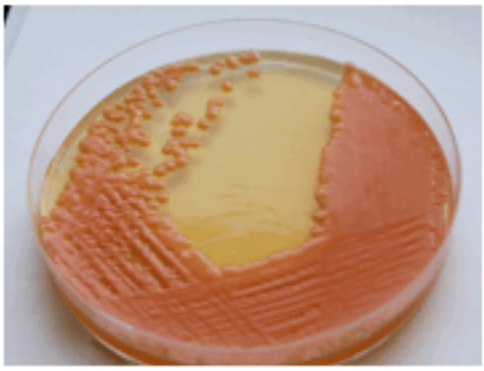

(b)

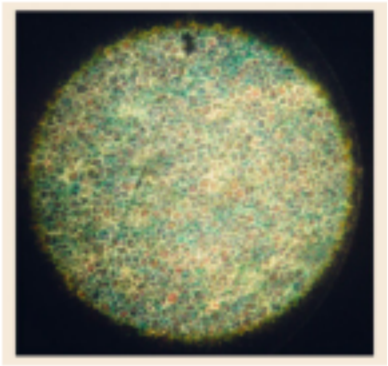

(c)

Figure S1. Colonies of *R. diobovata* DSBCA06 in YNB **(a)** and GYP **(b)** media; the same yeast cells observed at optic microscope **(c)**.

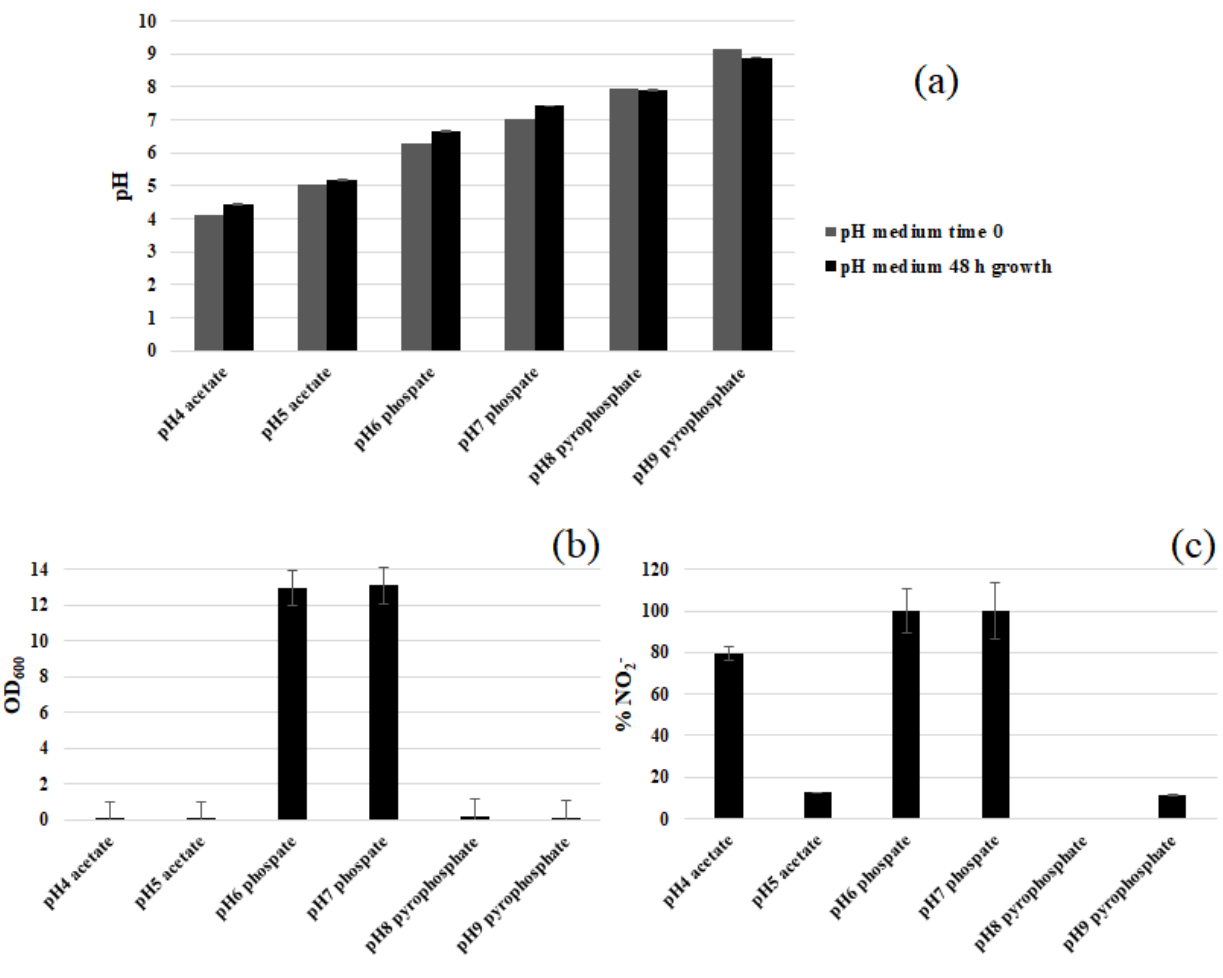

Figure S2. Influence of pH on *R. diobovata* DSBCA06 growth. In panel (**a**) initial and final pH values tested. Panels (**b**) and (**c**) report the corresponding growth and nitrite removal 48 h.

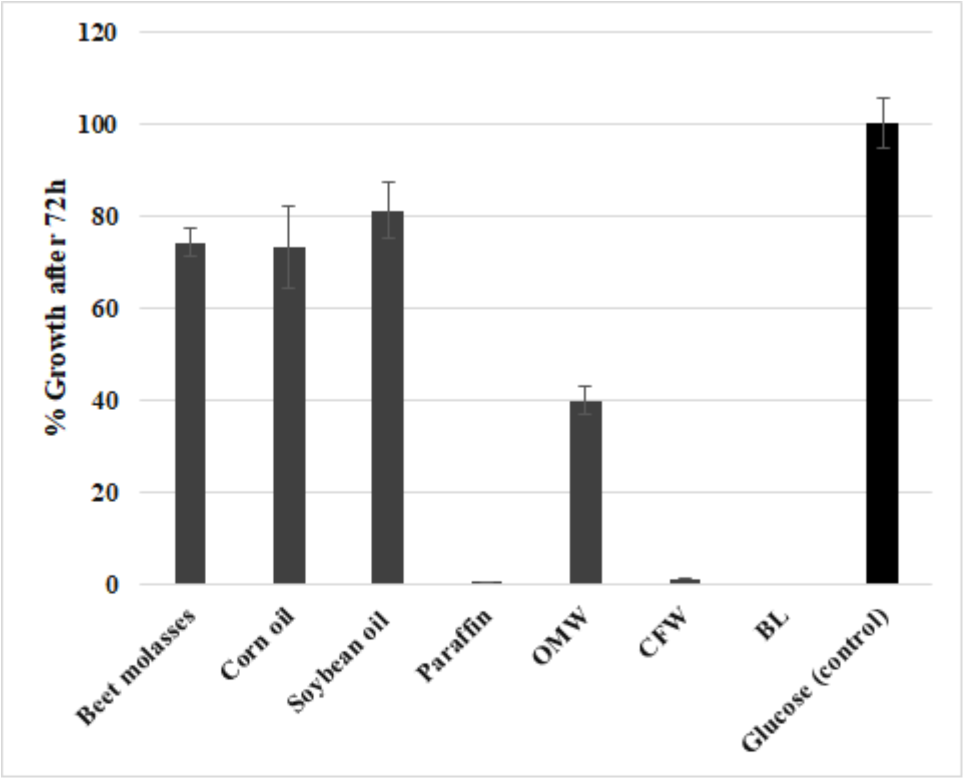

Figure S3. *R. diobovata* DSBCA06 growth in YNB using different renewable wastes as carbon sources. (OMW: olive mill wastewater; CFW: cork factory wastewater; BL: neutralized black liquor).
